# Weighted cart pull: A novel outcome measure for sustained motor function in mice

**DOI:** 10.1113/EP092658

**Published:** 2025-03-28

**Authors:** Charles D. Brennan, Nathan R. Kerr, Jose A. Viteri, Zachary Williard, Harper Snyder, Gabriella Meier, Sam Cairns, Fereshteh B. Darvishi, Anna R. Dashtmian, Peter J. Moore, Sindhuja N. Ayyagari, Meifang Wang, W. David Arnold

**Affiliations:** ^1^ School of Medicine, University of Missouri Columbia Missouri USA; ^2^ Tom and Anne Smith MD/PhD Program, University of Missouri Columbia Missouri USA; ^3^ Department of Physical Medicine and Rehabilitation, School of Medicine University of Missouri Columbia Missouri USA; ^4^ NextGen Precision Health University of Missouri Columbia Missouri USA

**Keywords:** ageing, motor function, power, sarcopenia, weakness

## Abstract

Sarcopenia, the pathological age‐related decline in muscle mass and strength, compromises independence and quality of life in older adults. Currently, no effective treatments are available. To enhance translational research using aged mouse models, we developed and validated the weighted cart pull (WCP) as a novel assessment of sustained motor function. The WCP test involved attaching a weighted cart to the tail of a mouse as it climbed a ramp to a ‘resting house’. Mass was increased incrementally until failure, defined as either five consecutive hindquarter pokes without progress or sliding backwards. In experiment 1, reliability (inter‐ and intra‐rater) was evaluated in middle‐aged mice (9 months, *n* = 10, 50% female). In experiment 2, young (*n* = 8, 50% female) and old (*n* = 8, 50% female) mice were tested on WCP, all‐limb grip and rotarod. In experiment 3, middle‐aged mice (7–9 months, *n* = 20, 50% female) underwent behavioural tests, in vivo electrophysiology and muscle physiology to correlate WCP with assessments of neuromuscular function. WCP showed high intra‐rater repeatability [intraclass correlation coefficient = 0.611, *P* = 0.018, 95% confidence interval (CI) = (0.046, 0.885)]. WCP demonstrated phenotypic differences between young and old mice (Student's unpaired *t*‐test, *P* < 0.0001). WCP was significantly correlated with all‐limb grip [Pearson's *r* = 0.5820, *P* = 0.0071, 95% CI = (0.1878, 0.8147)], percentage decrement upon repetitive nerve stimulation at 50 Hz [Pearson's *r* = 0.4613, *P* = 0.0468, 95% CI = (0.008973, 0.7569)], twitch torque [Pearson's *r* = 0.6241, *P* = 0.0033, 95% CI = (0.2509, 0.8358)], tetanic torque [Pearson's *r* = 0.5100, *P* = 0.0216, 95% CI = (0.08718, 0.7771)] and bilateral gastrocnemius and soleus muscle mass [Pearson's *r* = 0.5878, *P* = 0.0064, 95% CI = (0.1964, 0.8177)]. The WCP provides a cost‐effective, comprehensive measure of strength and sustained motor function, improving preclinical assessments.

## Abstract (highlights)

- **What is the central question of this study?**
  To what degree does the novel outcome measure, weighted cart pull, show validity in determining phenotypic differences in sustained motor function?
- **What is the main finding and its importance?**
  The weighted cart pull provides an inexpensive outcome measure for sustained motor function that is positively correlated with other behavioural tests of motor function, in vivo electrophysiology, in vivo muscle physiology and muscle wet masses. This assay can be used as a primary or additional outcome to assess differences in sustained motor function in preclinical studies.

## 1 INTRODUCTION

Skeletal muscle weakness and failure are defining characteristics of ageing and neuromuscular disorders, leading to significant impairments in mobility, independence and overall quality of life (Sayer et al., 2024; Seidler et al., 2010). Sarcopenia, the pathological loss of muscle mass and function, is associated with significant increases in all‐cause mortality (Ekram et al., 2021). When quantifying neuromuscular function, strength and power are related but distinct aspects of performance, each with unique implications for ageing and neuromuscular disease (Knuttgen & Kraemer, 1987; Reid & Fielding, 2012). Strength reflects the maximum force that a muscle can generate, whereas power combines force and velocity to measure the ability to perform dynamic and repetitive movements (Knuttgen & Kraemer, 1987). Although strength is crucial for basic activities, such as lifting or maintaining posture, power is equally important for tasks requiring dynamic and sustained effort, such as walking, climbing stairs or recovering from a loss of balance. Importantly, power and sustained motor output decline more rapidly than strength with ageing, making it a more sensitive indicator of functional impairment (Bårdstu et al., 2022; Reid & Fielding, 2012).

Preclinical mouse models are essential for investigating disease mechanisms and developing treatments for neuromuscular dysfunction. Behavioural assays of motor function are widely used to quantify disease phenotypes and evaluate treatment responses in mouse models. For instance, grip‐strength testing is a reliable method for measuring maximum force across various mouse models, including those studying ageing (Owendoff et al., 2023). However, the accuracy of grip‐strength measurements can be influenced by the experimental technique, such as mouse handling and the rate of pull during testing, introducing variability or bias (Montilla‐García et al., 2017; Owendoff et al., 2023). Despite its utility, grip strength has limitations as a comprehensive measure of motor function. It assesses only peak force, failing to capture power, which is a crucial measure of dynamic muscle performance. Power, which reflects both sustained muscle strength and contraction speed, is especially relevant in ageing and sarcopenia owing to its strong correlation with functional tasks such as rising from a chair, climbing stairs and preventing falls (Bean et al., 2002a, 2002b, 2004; Reid & Fielding, 2012). Another commonly used test, the rotarod, evaluates coordination and balance (Brooks et al., 2012). However, the rotarod assay also has limitations. Smaller mice might have an advantage owing to a smaller proportion of their body hanging over the edge of the rotating rod; a ceiling effect occurs when some mice outperform the maximum speed settings of the test; and mice can jump off owing to the distance of the rod from the ground (Deacon, 2013).

To address limitations of prior behavioural tests of motor function and, specifically, the lack of assessments of sustained motor function (e.g. power), we developed the weighted cart pull (WCP) test. The design of the WCP was adapted from a progressive resistance‐exercise method previously developed and reported by Zhu et al. (2021), which involved mice pulling a loaded cart as a form of resistance training. We modified the approach to include the use of an inclined ramp (9° incline compared with the horizontal plane), wire lining for increased grip and easy cleaning between trials, and low‐friction tracks to guide the weighted cart that was pulled by the mouse. In a series of three experiments, we sought to investigate the validity of the WCP. In experiment 1, we investigated intra‐ and inter‐rater reliability of this outcome measure, which showed good reliability. In experiment 2, we tested the ability of the WCP to detect age‐related loss of motor function by comparing results in young versus aged wild‐type mice and showed robust reduction of the maximum mass pulled in aged mice. In experiment 3, we examined the correlation between WCP results and outcomes from previously established behavioural assays, in addition to other measures of neuromuscular function, identifying significant relationships. By providing an accurate, inexpensive and reliable method for quantifying muscle function, the WCP offers a valuable tool for advancing research in ageing and neuromuscular disease.

## 2 MATERIALS AND METHODS

### 2.1 Ethical approval

All animal procedures were approved by University of Missouri Institutional Animal Care and Usage Committee (protocol 39905) and in accordance with the US federal guidelines of the Animal Welfare Act. All experimenters were trained and approved to work with laboratory animals. The authors understand the ethical principles related to scientific publishing and affirm that the research reported here complies with the journal's animal ethics checklist.

### 2.2 Experimental overview

**FIGURE 1:**
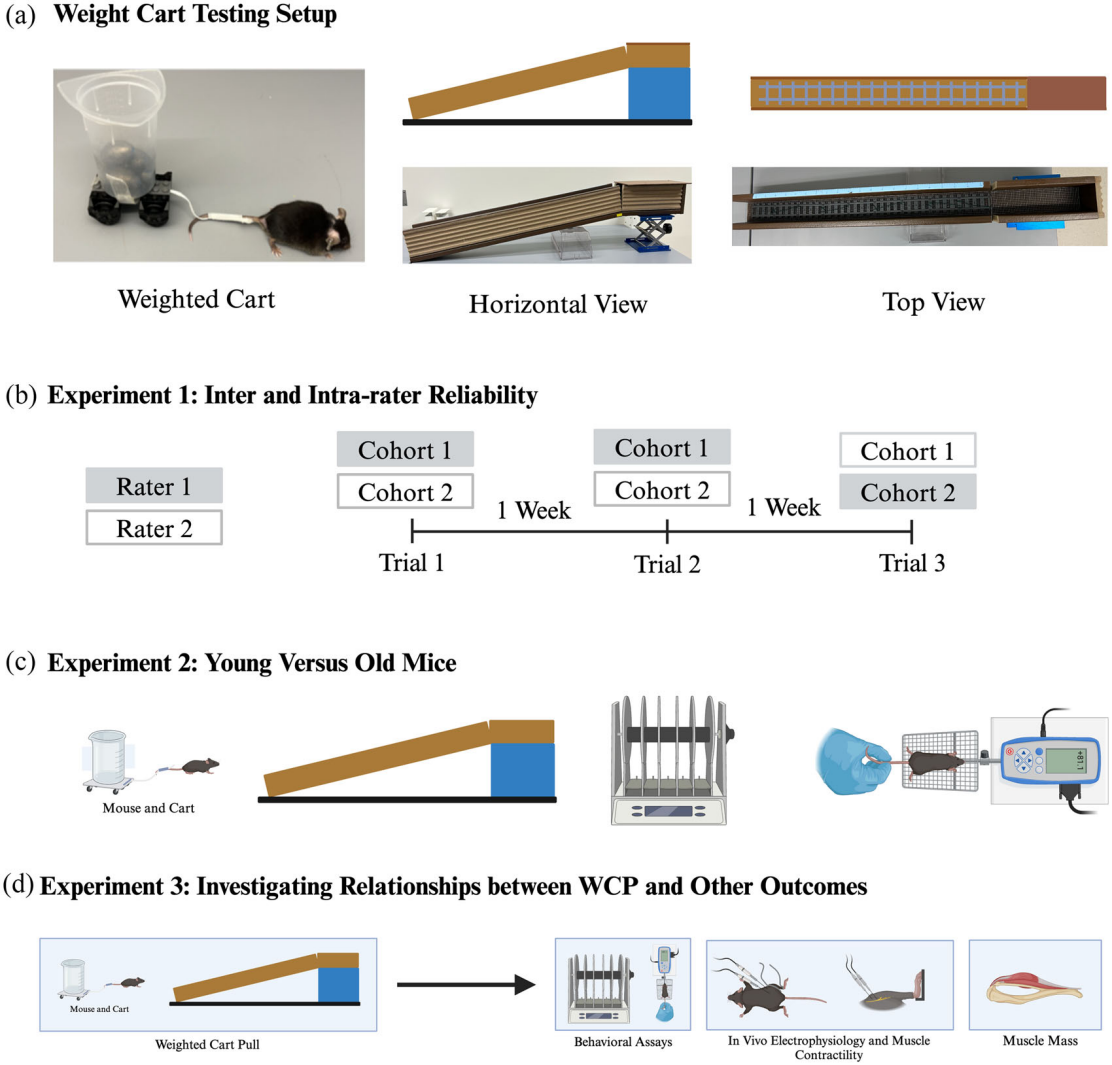
Study overview and design of the WCP apparatus. (a) Weighted cart pull testing set‐up. The mouse is attached to the weighted cart with tape. The inclined ramp apparatus is shown in horizontal view and without the resting house cover in top view. (b) Experiment 1: Intra‐ and inter‐rater variability. Mice tested by rater 1 are in the grey coloured square. Mice tested by rater 2 are in the white square with a grey outline. Cohorts 1 and 2 were tested by raters 1 and 2, respectively, for the first two trials. For the third trial, cohort 1 is tested by rater 2 and cohort 2 by rater 1. (c) Experiment 2: Testing WCP as an outcome measure. Young and old mice are tested on all‐limb grip, rotarod and WCP. (d) Experiment 3: Comparing WCP with other measures. Apparatus diagram with horizontal view and the cart attached to the mouse's tail. Behavioural assays (rotarod and all‐limb grip) along with in vivo electrophysiological and muscle physiological measures and gastrocnemius and soleus wet masses were also collected. Abbreviation: WCP, weighted cart pull.

In experiment 1, intra‐ and inter‐rater reliability were analysed for the WCP (Figure 1b). Two cohorts of mice were tested by two independent raters on WCP. Trials were separated by 1 week. Each cohort was tested only once per week by one of the raters. One week before trial 1, the mice were trained on WCP by a third independent rater to determine the ‘average maximum mass pulled’ per body mass for each cohort of mice. The ‘average maximum mass pulled’ was used to create customized mass progressions for each mouse, ensuring equal performance during runs up the ramp and controlling for fatigue. For trials 1 and 2, raters tested the same respective cohort for intra‐rater variation. In trial 3, cohorts were switched and compared with trial 2 for inter‐rater variation. For each successive trial, raters were blinded to previous trial results.

Furthermore, this study aimed to investigate the capacity of the novel outcome measure, WCP, to phenotype sustained motor function accurately in young and old mice. In experiment 2, a second group of young and old mice was evaluated on the WCP and standard measures of motor function, all‐limb grip and the rotarod (Figure 1c).

In experiment 3, a third group of mice was used to compare WCP with a battery of neuromuscular tests (Figure 1d). These included other behavioural assessments: grip and rotarod; in vivo electrophysiological measurements; in vivo muscle physiology; and muscle wet masses. Results were correlated to determine the predictive ability of the WCP for muscle form and function.

### 2.3 Animals

For experiment 1, middle‐aged wild‐type C57BL/6N mice (9 months old, *n* = 10, 50% female) were used. In experiment 2, young (3 months old, *n* = 8, 50% female) and old (28 months old, *n* = 8, 50% female) wild‐type C57BL/6N mice were used to test the efficacy of the WCP and its comparison to other measures. The rationale for these ages is based on prior work demonstrating a phenotypic difference in motor function in the grip and rotarod tests (Ge et al., 2016; Shoji et al., 2016).

Mice were bred and raised in sterile conditions with a 12 h–12 h light–dark cycle and free access to food and water (temperature 23°C–25°C, humidity < 10%). Mice were killed under ketamine and xylazine general anaesthesia (100 mg/kg ketamine and 7.5 mg/kg xylazine) by cervical dislocation. The gastrocnemius and soleus were removed from the mice bilaterally and weighed. Additionally, in experiment 3, 7‐ to 9‐month‐old (*n* = 20, 50% female) wild‐type C57BL/6N mice were used. Both sexes were as balanced as possible in each group of mice.

### 2.4 Ramp system

The custom‐built apparatus for the ramp strength‐training model is shown (Figure 1a) and included a 120‐cm‐long inclined ramp and a 50 cm resting house, covered with a 50 cm × 20 cm plastic board, 18.7 cm off of the ground to create a 9° angle that was built using impermeable plastic lumber (Fibron, Home Depot, MO, USA). The resting house was placed on a metal platform with an adjustable height. One‐quarter inch (0.635 cm) chicken wire was cut to the length of the track and the ‘resting house’. The width of the chicken wire was cut to be 2 cm greater than the width of the track. The chicken‐wire grip was then bent along each length towards the same side at a 90° angle by 1 cm such that the width of the grip fitted in the track and resting house. A plastic toy track (BroTex Brick Pack) was tied down to the centre of the chicken‐wire grip with additional wire.

### 2.5 Weighted cart and track design

A 4.8 cm × 6.4 cm cart, also custom‐built and shown in Figure 1a, was constructed with two sets of LEGO Wheels for RC Train (LEGO) and two 2 × 8 LEGO Parts and Pieces (LEGO). One end of a 15 cm length of 0.3175‐cm‐wide Cotton Umbilical Tape (Somerville, NJ, USA) was attached between the two 2 × 8 LEGOs and the other was attached to the tail of a mouse using 2 cm of transparent tape (Curad, NON270201). A plastic 100 mL beaker (height, 7.0 cm; diameter, 5.5 cm) was secured onto the top of the wheelbase with three 5‐cm‐long strips of transparent tape. These pieces assembled are the ‘unweighted cart’, which totalled 32 g. Steel masses were added as needed to create the ‘weighted cart’.

### 2.6 Weighted cart pull testing

#### 2.6.1 Familiarization/training

Mice were familiarized with the process of moving up the ramp tied to the ‘unweighted’ cart before mass was added progressively on subsequent runs. The added mass was determined for each mouse individually. One week before the testing day, mice were given a training day. This training day was to familiarize the mice with the track, to help eliminate novelty effects and to determine the ‘average maximum pull’ for the testing day. The ‘average maximum mass pulled’ was used with the mouse's body mass to tailor each mass regimen individually. Mice were grouped together, regardless of age and genotype, and the results of this training day were averaged. Each mouse consecutively pulled increasing mass until it failed to make it to the resting house. Failure was defined as a mouse not progressing for 5 s or sliding backwards after a poke was received. A poke perpendicular to the incline of the track was given on the hindquarters of the mouse if the mouse stopped on the track for >5 s. The first run consisted of only the unweighted cart. The next runs had totals of 100, 200, 250, 300 g of mass added to the unweighted cart. After 300 g of additional mass, 10.0 g of mass was added to each consecutive run. At failure, the cart was gently pushed behind the mouse, ensuring that the mouse traversed the whole length of the ramp. When a mouse failed to run the full length of the track, the previous successful mass was recorded and normalized to body mass, and the average for each group was considered the ‘average maximum pull’.

#### 2.6.2 Testing

For the WCP assay, mass was as follows: unweighted cart, 50% of average maximum pull, 80% of average maximum pull, 100% of average maximum pull, 115% of average maximum pull, 130% of average maximum pull and afterwards increasing by 25 g after every successful run beyond 130% of the average maximum pull. This was to ensure that mice were running a similar number of times up the track to control for fatigue. If the mice failed to pull 50% of the average maximum pull, then subsequent runs dropped 10% of their average maximum pull (40%, 30%, 20% and 10%) until mice were able to pass a run. A video of a successful trial is provided (Supplemental Video 1). Once mice reached failure, an example is provided (Supplemental Video 2), if they were at ≤130% of their average maximum pull, then they were run again with 25 g less than their failure run to improve resolution of their maximum pull mass. Between runs, the mice were allowed 2 min of rest.

The use of individual training values and subgroup training averages was considered in addition to the use of the ‘average maximum pull’, but these alternative methods for generating testing regimens inadvertently stratified the mice based on their training performance. Mice that underperformed during training would have been subjected to more repetitions and longer testing sessions; thus, they would be likely to be subjected to increased fatigue, potentially skewing results. The application of the group average, ‘average maximum pull’, provided consistent and equitable workloads across all animals, reducing the variability introduced by training performance while still accounting for differences in body size.

### 2.7 All‐limb grip strength

Grip experiments were performed in a similar manner to our prior work (Owendoff et al., 2023). Mice were held by the tail and placed on the ‘all‐limb’ grip attachment. A single rater performed all grip measurements at 10.00–12 h, 30 min after the WCP test. Mice were allowed to grip the attachment with all four paws and were then pulled by the tail parallel to the floor directly away from the force transducer. The grip recorded for each mouse was the average of three trials.

### 2.8 Rotarod

Rotarod (LE8205, Panlab Harvard Apparatus) was performed at 09.30–11:30 h. Mice were placed on the resting rod of the rotarod machine for 1 min. Mice were warmed up on the rotarod for 1 min at a constant speed of 4 rpm. Mice were allowed 1 min of rest on the resting rod. The rotarod was then set at a constant speed of 4 rpm for 30 s. After 30 s, a constant ramp from 4 to 40 rpm over 60 s was run, and the duration on the rod was recorded. Mice were given 1 min of rest on the resting rod, then the test was repeated twice. The average of these three trials was taken as the final rotarod measurement for each mouse.

### 2.9 in vivo electrophysiology

Similar to our prior work, in vivo motor unit electrophysiology was performed on the Sierra Summit EMG system (Cadwell Sierra Summit, Kennewick, WA, USA) with the amplifier settings of high‐pass filter at 10 kHz, low‐pass filter at 10 Hz and notch filter at 60 Hz (Chugh et al., 2020; Sheth et al., 2018). A pair of 28‐gauge monopolar needle electrodes (Teca, Oxford Instruments Medical, NY, USA) were used for nerve stimulation. Steel ring electrodes (Alpine Biomed, Skovlunde, Denmark) were used for muscle recordings. Mice were anaesthetized via intraperitoneal injection of ketamine and xylazine (Covetrus, Portland, ME, USA; 100 mg/kg ketamine and 7.5 mg/kg xylazine). Proper anaesthesia depth were determined by lack of response to a toe pinch. Hindlimbs were taped down in prone extension using adhesive tape (Transpore) on a heating pad set to 36.5°C. Two ring electrodes were placed on the right leg of the mouse, with the recording electrode around the belly of the gastrocnemius muscle and the reference electrode around the mid‐metatarsal. Measurements recorded included compound muscle action potential (CMAP), motor unit number estimation (MUNE), single motor unit potential (SMUP) and percentage decrement upon repetitive nerve stimulation at 50 Hz (RNS). Briefly, CMAP was measured by supramaximal stimulation of the right sciatic nerve (<10 mA constant current intensity, 0.1 ms pulse duration and 20 mV sensitivity), where peak‐to‐peak values were recorded. The SMUP was measured by incrementally increasing submaximal stimulations (from 50 to 500 µV sensitivity) and averaging the steps to determine the single motor unit potential. The MUNE is equivalent to the CMAP amplitude peak to peak divided by the average SMUP amplitude peak to peak. The RNS was measured by inducing a train of 10 supramaximal stimuli, the same to generate CMAP amplitude, to the sciatic nerve at 10, 20, 30, 40 and 50 Hz. Ten seconds were allowed between each train of 10 stimuli. Differences in CMAP amplitude between the first stimulation and the 10th stimulation were divided by the amplitude of the first response and recorded as the decrement (Padilla et al., 2021).

### 2.10 Muscle physiology

The experimental procedure was performed as previously described (Sheth et al., 2018). Mice were placed supine on a heating pad at 36.5°C. The right hind paw was taped to the rotating foot plate. Clamps were applied to the femoral condyles to prevent lateral movement. The stage was moved to ensure a 90° angle at the ankle. Two stimulating electrodes were inserted subcutaneously at the proximal, medial right leg to activate the tibial branch of the sciatic nerve. The stimulus was increased to determine the maximum stimulus intensity, which, for subsequent experiments, was increased to 120%–150% of that value. After a single supramaximal 0.2 ms square‐wave pulse, twitch torque was recorded. After a 500 ms train of the 0.2 ms stimuli at 150 Hz, the tetanic torque was measured.

### 2.11 Muscle mass

Immediately after the mice were killed, the gastrocnemius and soleus muscles were dissected. The skin was removed from the lower limb. The hamstring was partly cut to expose the upper gastrocnemius. Blunt dissection was used to remove the hamstring. A probe was placed under the Achilles tendon and was moved up against the tibia proximally. Scissors were used to cut the Achilles tendon and the upper tendons of the gastrocnemius and soleus. To standardize muscle extraction, ∼1 mm of tendon was preserved on either end of the gastrocnemius and soleus during extraction.

### 2.12 Statistical analysis

Group data are represented as the mean ± SD. Statistics were calculated using Prism v.10.3.1 GraphPad and IBM SPSS. Data were analysed and compared using Student's unpaired *t*‐test, Welch's *t‐*test (unpaired *t*‐test with Welch's correction), coefficient of variation (CV), simple linear regression with Pearson's correlation coefficient [95% confidence interval (CI) shown with dashed lines] and single‐measures two‐way random effect intraclass correlation coefficient (ICC). Coefficients of variation were calculated for each mouse individually, then averaged together. In all cases, significance was considered at *P* < 0.05.

## 3 RESULTS

### 3.1 Experiment 1: Weighted cart pull design and test–retest reliability

In an attempt to adapt the method of Zhu et al. (2021) to an outcome measure, different modifications were compared. The half‐Atwood pulley system and the WCP on an inclined ramp were the two most promising designs; however, the inclined ramp system was chosen based on our observation of superior voluntary participation with WCP relative to the pulley system (data not shown). After the inclined ramp design was selected, the former grip of a foam kitchen liner was exchanged for chicken wire to provide better traction for the mice and easier cleaning between trials.

**FIGURE 2:**
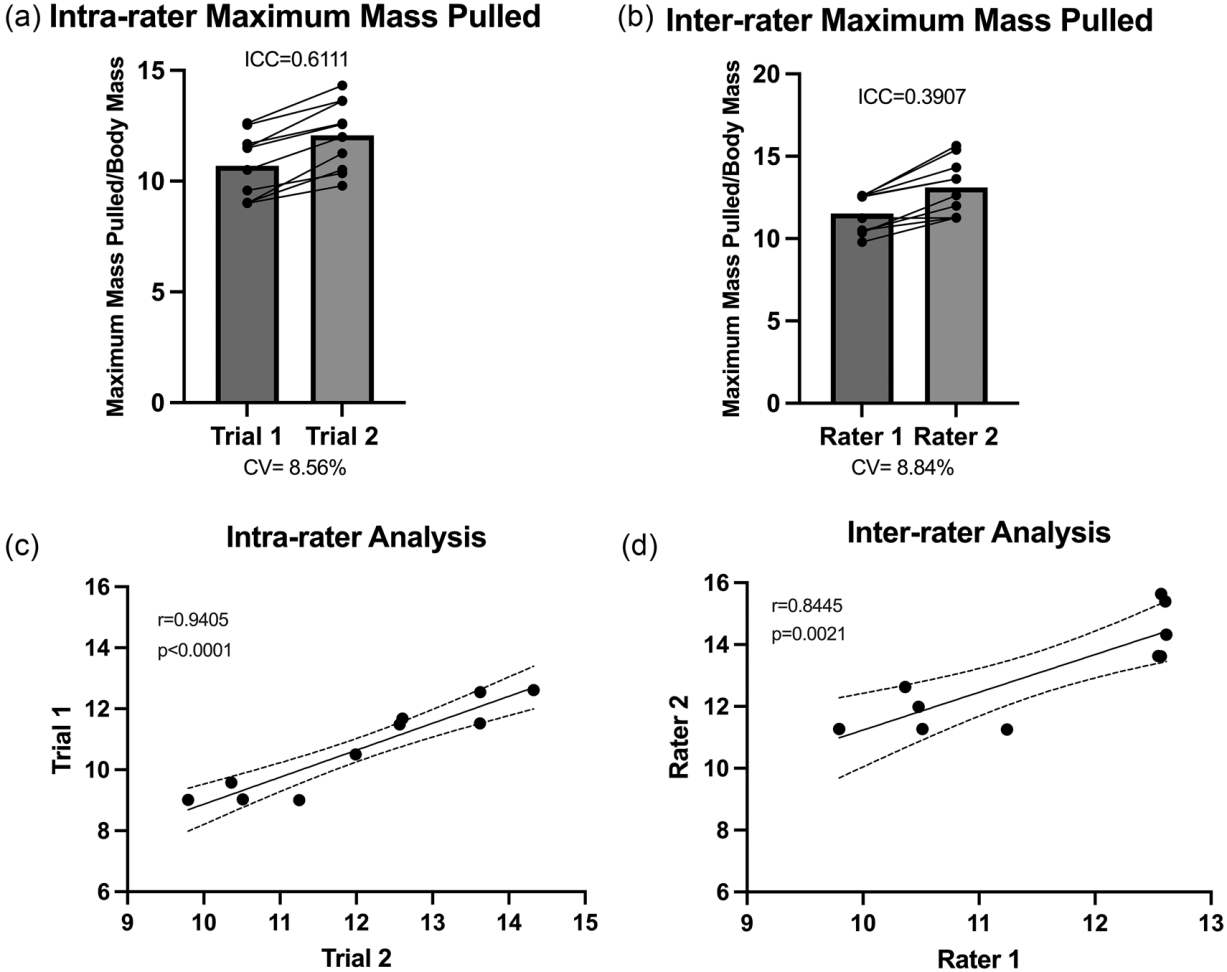
The WCP demonstrated high intra‐rater and moderate inter‐rater reliability. Two naive raters were given training on WCP techniques. Cohort 1 was rated by rater 1 for the first 2 weeks. Cohorts were crossed over for the third week, and cohort 1 was rated by rater 2. (a) Intra‐rater WCP normalized to body mass [average CV = 8.56%; single measures ICC = 0.611, *P* = 0.018, 95% CI = (0.046, 0.885)]. (b) Inter‐rater WCP normalized to body mass [average CV = 8.84%; single measures ICC = 0.391, *P* = 0.107, 95% CI = (−0.247, 0.801)]. (c) For the intra‐rater analysis, trial 1 and trial 2 are significantly correlated [Pearson's *r* = 0.9405, *P *< 0.0001, 95% CI = (0.7622, 0.9862)]. (d) For the inter‐rater analysis, rater 1 and rater 2 show a significant correlation [Pearson's *r* = 0.8445, *P* = 0.0021, 95% CI = (0.4587, 0.9624)]. Abbreviations: CI, confidence interval; CV, coefficient of variation; ICC, intraclass correlation coefficient; WCP, weighted cart pull.

To assess repeatability of the test, inter‐ and intra‐rater reliability was assessed with repeated trials of the WCP. Two laboratory technicians with minimal experience of WCP were trained. Next, the raters were each assigned a cohort of mice (cohort 1: 9 months old, *n* = 5, 60% female; and cohort 2: 9 months old, *n* = 5, 40% female), with which they performed WCP once a week for 2 weeks. The results from weeks 1 and 2 were compared and showed a strong intra‐rater repeatability [Figure 2a,c; average CV = 8.56%; single measures ICC = 0.611, *P* = 0.018, 95% CI = (0.046, 0.885); Pearson's *r* = 0.9405, *P *< 0.0001, 95% CI = (0.7622, 0.9862)]. Then, in week 3 the cohorts were exchanged and tested by the other rater to assess inter‐rater reliability. The results from week 2 and week 3 were compared and demonstrated moderate inter‐rater repeatability [Figure 2b,d; average CV = 8.84%; single measures ICC = 0.391, *P* = 0.107, 95% CI = (−0.247, 0.801); Pearson's *r* = 0.8445, *P* = 0.0021, 95% CI = (0.4587, 0.9624)]. Mice showed no changes in body mass over the course of the three trials (data not shown).

Altogether, these results show that the WCP shows strong intra‐rater consistency and moderate inter‐rater consistency.

### 3.2 Experiment 2: Weighted cart pull as an outcome measure of age‐related loss of motor function

**FIGURE 3:**
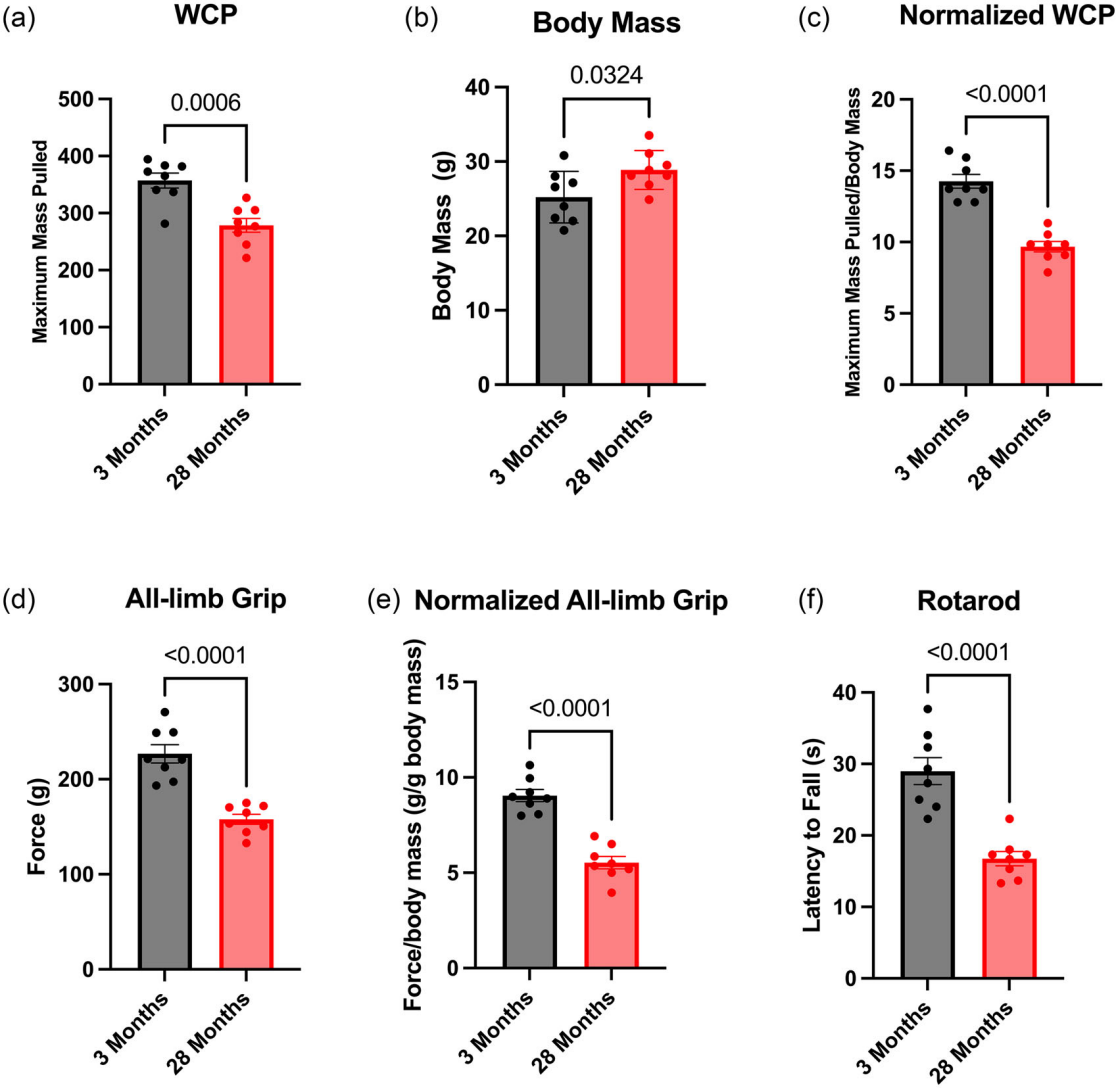
The WCP demonstrated phenotypic differences in 3‐ and 28‐month‐old mice similar to all‐limb grip and rotarod. (a) Three‐month‐old mice exhibited increased maximum mass pulled compared with 28‐month‐old mice (Student's unpaired *t*‐test, *P* = 0.0006). (b) Twenty‐eight‐month‐old mice had significantly greater body mass compared with 3‐month‐old mice (Student's unpaired *t*‐test, *P* = 0.0324). (c) Three‐month‐old mice exhibited increased maximum mass pulled normalized to body mass compared with 28‐month‐old mice (Student's unpaired *t*‐test, *P *< 0.0001). (d) Three‐month‐old mice exhibited significantly increased all‐limb grip compared with the 28‐month‐old mice (Student's unpaired *t*‐test, *P *< 0.0001). (e) Three‐month‐old mice exhibited significantly increased all‐limb grip normalized to body mass compared with 28‐month‐old mice (Student's unpaired *t*‐test, *P *< 0.0001). (f) Time on the rotarod was significantly longer for the 3‐ compared with 28‐month‐old mice (Student's unpaired *t‐*test, *P *< 0.0001). Abbreviation: WCP, weighted cart pull.

To explore the capacity of the WCP to detect age‐related decline in motor function, the test was performed on a cohort of young (3‐month‐old) and old (28‐month‐old) mice. Young mice pulled significantly more mass than the old mice (Figure 3a; Student's unpaired *t‐*test, *P* = 0.0006). These young mice had significantly lower body mass than the old mice (Figure 3b; Student's unpaired *t*‐test, *P* = 0.0324); thus, WCP normalized to body mass was analysed, and young mice pulled significantly more mass on WCP than old mice when normalized to body mass (Figure 3c; Student's unpaired *t*‐test, *P *< 0.0001).

Mice were also tested on standard metrics of motor function: all‐limb grip strength and rotarod. Old mice showed decreased maximal force output in the all‐limb grip strength compared with young mice both not normalized to body mass and normalized to body mass (Figure 3d; Student's unpaired *t*‐test, *P* < 0.0001; and Figure 3e; Student's unpaired *t*‐test, *P *< 0.0001). Additionally, young mice performed significantly better (evidenced by a greater latency to fall) on the rotarod compared with old mice (Figure 3f; Student's unpaired *t*‐test, *P *< 0.0001).

Overall, these findings indicate that the WCP can detect a phenotypic difference between young and old mice.

### 3.3 Experiment 3: Comparison of weighted cart pull with other metrics of motor function

**FIGURE 4:**
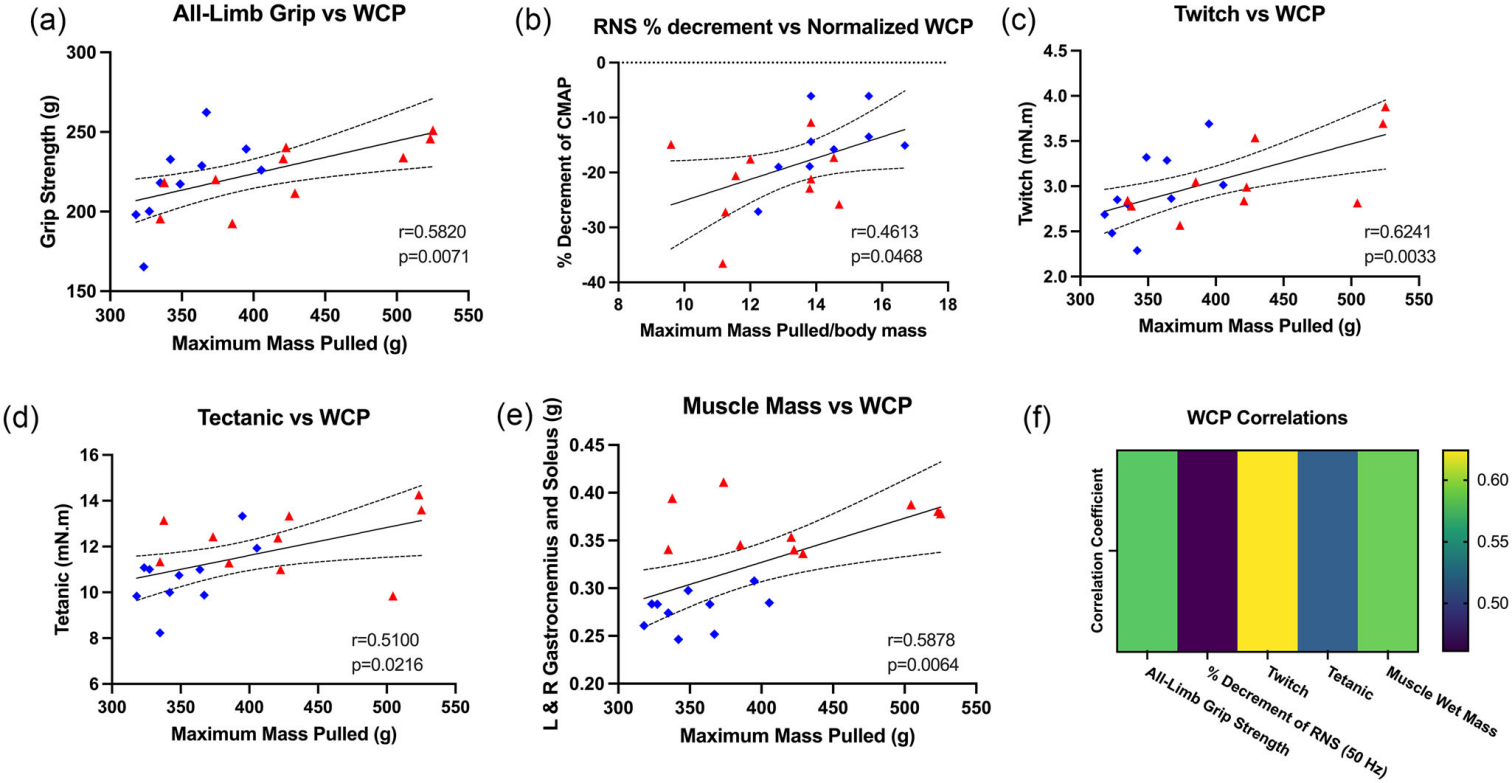
The WCP is correlated with behaviour, in vivo electrophysiology, in vivo muscle physiology and anatomical measures of motor function. WCP is normalized to body mass only for in vivo electrophysiology correlations (percentage decrement with RNS). Male mice are represented as red triangles and female mice as blue diamonds. (a) All‐limb grip was significantly correlated with WCP [Pearson's *r* = 0.5820, *P* = 0.0071, 95% CI = (0.1878, 0.8147)]. (b) Percentage decrement of RNS at 50 Hz was significantly correlated with WCP normalized to body mass [Pearson's *r* = 0.4613, *P* = 0.0468, 95% CI = (0.008973, 0.7569)]. (c) Twitch torque showed a significant correlation with WCP [Pearson's *r* = 0.6241, *P* = 0.0033, 95% CI = (0.2509, 0.8358)]. (d) Tetanic torque was significantly correlated with WCP [Pearson's *r* = 0.5100, *P* = 0.0216, 95% CI = (0.08718, 0.7771)]. (e) Bilateral gastrocnemius and soleus muscle mass was significantly correlated with WCP [Pearson's *r* = 0.5878, *P* = 0.0064, 95% CI = (0.1964, 0.8177)]. (f) Heat map of absolute values for Pearson correlation coefficients. Abbreviations: CI, confidence interval; L, left; R, right; RNS, repetitive nerve stimulation; WCP, weighted cart pull.

After demonstrating the ability of the WCP to detect age‐related deficits in motor function, we aimed to determine whether our novel assessment is correlated with other established measures of motor function. Thus, a new cohort of mice (*n* = 20, 50% female) ranging from 7 to 9 months old were tested on WCP and a battery of neuromuscular assessments, including all‐limb grip, rotarod, in vivo electrophysiology, in vivo muscle physiology and muscle wet mass collection. These other established methods of neuromuscular function were then correlated with WCP (Figure 4). All‐limb grip strength demonstrated a significant positive correlation with WCP [Figure 4a; Pearson's *r* = 0.5820, *P* = 0.0071, 95% CI = (0.1878, 0.8147)]. Yet, WCP normalized to body mass showed a non‐significant positive correlation with rotarod [data not shown; Pearson's *r* = 0.3161, *P* = 0.1745, 95% CI = (−0.1470, 0.6655)]. These results support the WCP assay as a comparable assessment of motor function to the more established all‐limb grip strength test.

In addition to behavioural measures of motor function, we measured in vivo neuromuscular electrophysiology and muscle physiology in the same cohort of 7‐ to 9‐month‐old mice. Several physiological parameters showed significant correlation with the WCP test (Figure 4). From the in vivo electrophysiological measures, the percentage decrement of RNS at 50 Hz demonstrated a significant positive correlation with WCP [Figure 4b; Pearson's *r* = 0.4613, *P* = 0.0468, 95% CI = (0.008973, 0.7569)]. WCP did not show significant correlations with CMAP [data not shown; Pearson's *r* = −0.1679, *P* = 0.4791, 95% CI = (−0.5682, 0.2966)], SMUP [data not shown; Pearson's *r* = −0.1692, *P* = 0.4757, 95% CI = (−0.5691, 0.2954)] or MUNE [data not shown; Pearson's *r* = −0.09844, *P* = 0.6797, 95% CI = (−0.5184, 0.3598)].

Both measures of in vivo muscle physiology showed significant positive correlations with WCP: twitch torque [Figure 4c; Pearson's *r* = 0.6241, *P* = 0.0033, 95% CI = (0.2509, 0.8358)] and tetanic torque [Figure 4d; Pearson's *r* = 0.5100, *P* = 0.0216, 95% CI = (0.08718, 0.7771)]. These results suggest that WCP was strongly correlated with muscle output.

Furthermore, gastrocnemius and soleus muscles were harvested bilaterally, and masses were found to be significantly correlated with the WCP [Figure 4e; Pearson's *r* = 0.5878, *P* = 0.0064, 95% CI = (0.1964, 0.8177)].

These significant correlations are summarized in a heatmap (Figure 4f). Overall, these results support the ability of the WCP to assess motor function accurately.

## 4 DISCUSSION

This study introduces a new assay, the WCP test, as a method for assessing motor function in preclinical models. The WCP test demonstrated moderate inter‐ and strong intra‐rater reliability, with CVs and ICCs supporting its repeatability. Notably, the WCP detected significant age‐related deficits in muscle function, aligning with prior findings from all‐limb grip. Furthermore, the assay showed strong correlations with neuromuscular physiology and muscle mass measures, supporting its suitability as an outcome measure in a preclinical model of age‐related weakness.

### 4.1 Development and reliability of the weighted cart pull assay

The WCP was adapted from the progressive resistance exercise method described by Zhu et al. and optimized as an outcome measure through a series of modifications, implemented for specific reasons (Zhu et al., 2021). First, in our approach, an incline was added to provide constant tension of the cart on the tail of the mouse. Different inclines were tested using a fixed length of the track similar to that used previously (Zhu et al., 2021). The height of the track used was chosen such that mice could easily traverse the track while still allowing for enough mass to be pulled to remain a sensitive test. The chicken‐wire grip was added to give the mouse a better grip, to minimize slippage as a limiting factor for the performance of a mouse. Most importantly, the chicken wire allows for more thorough cleaning of the track. Finally, the Lego track and cart were used guide the cart in order to minimize friction between the cart and the walls of the ramp. These modifications of the previous model helped to optimize the measurement of motor function.

Both young and old mice tested showed a willingness to traverse the ramp (see Supplemental Videos 1 and 2). This intrinsic motivation might stem from several factors, such as the pull of the cart on the tail creating a pressure sensation that encourages the mouse to ascend the track to ‘escape’. Upon reaching the resting house, this sensation diminishes as the cart transitions to a horizontal plane, relieving tension on the tail of the mouse. The reward of entering the small, dark enclosure of the resting house might further motivate the mouse to traverse the uncovered track. Moreover, when the mouse fails to advance, a light ‘poke’ is delivered to the mouse to provide additional motivation. When mice reach failure, a majority will struggle for several seconds to advance, then release their grip on the track and allow the cart to pull them backwards, sliding down the track.

The WCP shows a strong intra‐rater reliability. Although inherent differences between rater assay handling are likely to influence some of the variability seen, owing to the experiment being performed over 2 weeks, true changes to the physical condition and performance of the mouse over time might also contribute to the differences between groups. To balance minimizing time‐related changes while reducing the potential training effects of repetitive testing, a 1 week interval was chosen between testing sessions.

### 4.2 The WCP as an outcome measure

After determining the repeatability of the WCP assay, we sought to determine its ability to detect phenotypic differences between young and old mice. This difference in WCP between young and old mice is consistent with the decreased strength in old mice compared with young mice (Ge et al., 2016; Shoji et al., 2016). Similar to established measures of motor function (all‐limb grip and rotarod), the WCP shows strong differences between young and old mice. As discussed previously, each of these behavioural assays assesses different aspects of motor function. Grip is a strong measure of peak motor output, rotarod incorporates balance and coordination with muscle function, and WCP measures sustained motor output. Used together, these tests can help to tease apart the multifactorial nature of functional strength.

### 4.3 Comparison with existing methods

Behavioural assays of motor function, such as grip strength and rotarod, are indispensable tools for studying muscle function, yet each has notable limitations. Grip strength, although measuring force directly, is subject to variability influenced by factors such as the motivation of the mouse and pain (Montilla‐García et al., 2017; Owendoff et al., 2023). Furthermore, grip strength assesses only peak force, making it particularly susceptible to variations in the speed at which the rater pulls the mouse from the grip meter. In contrast, the WCP requires sustained strength and has a more structured system for experimenter involvement than grip. Moreover, this sustained motor function is likely to be more relevant than peak force in clinical outcomes. Thus, sustained strength output might more accurately reflect the impact of ageing and disease in preclinical models and the efficacy of therapeutic interventions.

In contrast to grip, rotarod performance integrates several factors, including balance, coordination and endurance. Yet, several limitations exist. Larger mice have more difficulty remaining on the rod because a greater proportion of the body is hanging off the rod. Thus, balance becomes a more important confounder for larger mice, making it more difficult to draw conclusions about motor function when comparing them with smaller mice using the same rod size. The WCP addresses this limitation by minimizing the influence of mouse size by personalizing the assessment based on mouse body mass. Also, mice can turn around on the rotarod during the experiment, which can cause them to slip in this manoeuvre or, if they successfully turn around, undergo a completely different task, having to walk backwards. Mice, additionally, have a tendency to jump off the rotarod (Deacon, 2013). These limitations of the rotarod do not occur in the WCP experiment, in which the mice attempt to run away from the pull of the cart on their tails, and mice cannot ‘jump off’ early or ‘slip off’ while turning around.

Owendoff et al. (2023) have demonstrated all‐limb grip strength intra‐rater reliability in mice to have an ICC = 0.45, 95% CI = (0.02, 0.74) and CV = 8.6%. Our study shows that WCP has a higher intra‐rater reliability, based on ICC = 0.6111, with a similar variability, based on CV = 8.56%.

The WCP shows a significant correlation with grip strength, but not with the rotarod. This highlights the nuanced difference between what each assay tests. One explanation for this difference is that all‐limb grip strength is a more direct measure of strength than rotarod. Coordination and balance might influence the rotarod results more than the WCP and all‐limb grip assays. Its positive correlations with all‐limb grip supports WCP as a measure of strength output.

### 4.4 Physiological correlations

The WCP is closely correlated with other physiological assessments of neuromuscular function. Parameters including the percentage decrement during RNS (an assessment of neuromuscular junction transmission with nerve stimulation at 50 Hz) and measures of muscle contractile torque production (twitch and tetanic torque) were strongly correlated with WCP, underscoring its ability to reflect functional motor output. The association of the WCP test with RNS percentage decrement aligns with the concept of WCP measuring sustained motor function. Mice with less neuromuscular junction transmission failure with repetitive firing should demonstrate improved sustained motor output. These findings align with RNS testing results seen in patients with the prototypical neuromuscular junction disorder, myasthenia gravis (Tomschik et al., 2023). The correlations between WCP and contractile function (twitch torque and tetanic torque) contribute further to the argument for accurate assessment of motor function by the WCP. Interestingly, CMAP, SMUP and MUNE did not show significant correlations with WCP test, which might indicate that these metrics are less sensitive to sustained functional muscle output in the conditions tested. The positive correlation of WCP with the gastrocnemius and soleus muscle mass adds to the evidence supporting the WCP as an outcome measure of motor function. Although not powered to analyse sex differences in these correlations, a secondary analysis (data not shown) has demonstrated that all‐limb grip and twitch torque had the strongest correlations when sexes were separated. Moreover, all correlations when separated by sex showed trends in the same direction; some correlations were driven more by the females and others by the males.

### 4.5 Limitations and future studies

In the present study, we applied the WCP test to a model of age‐related loss of physical function. Future studies should focus on optimizing the WCP protocol for broader applicability, including its use in models of neuromuscular disease. Additionally, a simple cross‐sectional study design was implemented (only a single time point was assessed). Thus, longitudinal studies could provide insights into how WCP performs across the disease course, particularly in conditions where muscle weakness is progressive. We recently used this outcome measure in a preclinical study, demonstrating its effectiveness in assessing the impact of a serotonin 2c receptor, 5‐HT2C, agonist and antagonist on neuromuscular function in aged mice (Kerr et al., 2025). Additional use to evaluate therapeutic interventions in sarcopenia, cachexia and other neuromuscular conditions will further establish its utility as a preclinical tool.

Despite the natural inclination of mice to traverse the ramp, the WCP protocol includes a habituation phase. The training session, conducted 1 week before the study begins, aims to address the learning process of this protocol, with the training session 1 week before the start of the study as an attempt to account for this learning curve. Nonetheless, we still observed some improvement, either owing to improvement in absolute strength or owing to improved learning of the task. The former might be resolved by increasing the space between trails. The latter might be resolved by increased habituation or training sessions before testing. Yet, if testing is repeated too often, absolute muscle strength might also increase owing to a training effect. For our study, a minimum interval between trials of 1 week was set to minimize any training effect. A combination of more training sessions and increased time between the trials might further reduce the training effect seen. Like any behavioural assessment, the WCP is not immune to influences such as pain, fear and intrinsic motivation. Overhandling the mice might exacerbate these limitations. Other factors that might confound the WCP include lighting, sounds and smells.

Furthermore, a limitation of this study is the age range of the mice used for physiological correlations (experiment 3). The 7‐ to 9‐month‐old mice were chosen to establish baseline correlations between WCP performance and neuromuscular function in a cohort without significant age‐related decline. This approach allowed for the validation of the WCP as a reliable measure of sustained motor function in typical physiological conditions, providing broader applicability for general use in preclinical studies. Future experiments might investigate physiological correlations between WCP and various phenotypes, including ageing, sex differences and neuromuscular disease.

Future directions include using the WCP model as a progressive resistance exercise (PRE) model, similar to the work by Zhu et al. (2021) but with the included inclined ramp. Other PRE models in rats have used the vertical ladder climbing apparatus (Kelty et al., 2022; Souza et al., 2017). However, mice are likely to be less compliant than rats in performing the vertical ladder climb; thus, the WCP could be used to adapt this PRE training for mice. Importantly, the WCP test could be used as both the training system and an outcome measure in addition to other outcome measures assessed in this study (Zhu et al., 2021).

## 5 CONCLUSION

In conclusion, our study presents a promising and cost‐effective outcome measure with a strong ability to measure sustained functional strength and high repeatability. The validity of the outcome measure is supported by the significant correlations between WCP and other behavioural assessments, in vivo electrophysiology, muscle physiology and muscle mass. The WCP can augment the current measures of muscle strength and, like the work by Zhu et al. (2021), can even double as a model for progressive resistance exercise. Further studies are needed to show the efficacy of the WCP as a meaningful outcome measure in other preclinical mouse models.

## AUTHOR CONTRIBUTIONS

All experiments were performed at the University of Missouri, Columbia. Conceived and designed the research strategy: Charles D. Brennan and W. David Arnold. Designed and constructed WCP apparatus: Charles D. Brennan, Zachary Williard and Meifang Wang. Performed mouse colony management: Meifang Wang. Performed behavioural assays for WCP comparison with other outcome measures: Charles D. Brennan. Performed intraperitoneal ketamine/xylazine injections: Charles D. Brennan. Performed in vivo electrophysiology: Nathan R. Kerr and Charles D. Brennan. Performed in vivo muscle physiology: Jose A. Viteri. Performed gastrocnemius and soleus extraction: Charles D. Brennan. Performed intra‐ and inter‐rater reliability behaviour testing: Harper Snyder and Gabriella Meier. Wrote and edited manuscript: Charles D. Brennan, Nathan R. Kerr, Jose A. Viteri and W. David Arnold. Supervised the project: W. David Arnold. All authors approved the final version of the manuscript and agree to be accountable for all aspects of the work in ensuring that questions related to the accuracy or integrity of any part of the work are appropriately investigated and resolved. All persons demarcated as authors qualify for authorship, and all qualifying for authorship are listed.

## CONFLICT OF INTEREST

W. David Arnold has received grant funding from NMD Pharma, Avidity Biosciences, consulting fees from Avidity Biosciences, NMD Pharma, Dyne Therapeutics, Genentech, Design Therapeutics, Catalyst Pharmaceuticals and Insmed regarding therapeutic development for Duchenne muscular dystrophy, myotonic dystrophy type 1, and spinal muscular atrophy.

## Supporting information




**SUPPLEMENTAL VIDEO 1** Video of a 25‐month‐old mouse completing a run on weighted cart pull.

**SUPPLEMENTAL VIDEO 2** Video of 25‐month‐old mouse failure.

## Data Availability

Data underlying this study are available in the published article and in online Supplementary Material.
